# Paraneoplastic progressive encephalomyelitis with rigidity and myoclonus associated with monoclonal B-cell lymphocytosis in the setting of longstanding methotrexate use: case report

**DOI:** 10.3389/fimmu.2024.1436733

**Published:** 2024-10-16

**Authors:** Fangzhi (Frank) Jia, Mohammad Ruhul Amin, Gi Tae Kwon, Amir Mousapasandi, Pei Dai, Jed Kitson, Adrian Selim, Jerome Ip

**Affiliations:** ^1^ Department of Neurology, Nepean Hospital, Kingswood, NSW, Australia; ^2^ School of Clinical Medicine, St Vincent's Healthcare, Faculty of Medicine and Health, UNSW Sydney, Sydney, NSW, Australia; ^3^ Department of Immunology, Nepean Hospital, Kingswood, NSW, Australia; ^4^ Intravital Microscopy and Gene Expression (IMAGE) Lab, Garvan Institute of Medical Research, Darlinghurst, NSW, Australia; ^5^ Department of Haematology, Nepean Hospital, Kingswood, NSW, Australia; ^6^ Department of Neurology, The Sydney Adventist Hospital, Wahroonga, NSW, Australia; ^7^ Section of Neurology, Hornsby Hospital, Hornsby, NSW, Australia

**Keywords:** progressive encephalomyelitis with rigidity and myoclonus, glycine receptor antibody, monoclonal B-cell lymphocytosis, paraneoplastic neurological disorder, methotrexate-associated lymphoproliferative disorder

## Abstract

Progressive encephalomyelitis with rigidity and myoclonus (PERM) is a rare but debilitating disease within the stiff person syndrome (SPS) spectrum characterised by muscle rigidity, spasms, myoclonus, dysautonomia, and brainstem dysfunction. The exact pathogenetic mechanism is unclear, although there is an association with the presence of glycine receptor antibodies in serum and cerebrospinal fluid, and some cases are paraneoplastic. Here, we report a case of paraneoplastic, glycine receptor antibody-positive PERM associated with an otherwise subclinical monoclonal B-cell lymphocytosis (MBL) of the non-CLL phenotype, which may be, in turn, likely secondary to long-term methotrexate use [i.e., methotrexate-associated lymphoproliferative disorder (MTX-LPD)] or an underlying autoimmune disease. Treatment with multiple lines of initial induction immunomodulatory therapies, followed by maintenance rituximab, achieved long-term remission of the neurologic, haematological, and rheumatologic disease. This is, to our knowledge, the first reported association between PERM and MBL, or between PERM and MTX-LPD.

## 1 Introduction

Progressive encephalomyelitis with rigidity and myoclonus (PERM) is one of the stiff person-plus disorders and is distinctively characterised by the presence of brainstem dysfunction. Patients normally present with progressive muscular rigidity and spasms, myoclonus, brainstem signs, hyperekplexia, long-tract signs, and autonomic dysfunction. The disease often follows an aggressive course and carries a high likelihood of mortality and morbidity. Patients typically improve with immunomodulatory therapies, although complete neurologic recovery may not occur and there is a high relapse rate.

Since its initial description by Whiteley et al. in 1976 (1), historical cases of PERM were often attributed to glutamic acid decarboxylase 65 (GAD65) antibodies. In 2008, glycine receptor (GlyR) autoantibodies were first reported in a patient with PERM; since then, an increasing body of research has shown the pathogenic role of GlyR antibodies in PERM, and it is now accepted that GlyR antibodies (specifically targeting the α1 subunit of the receptor) are the hallmark of PERM, unlike stiff person syndrome (2–4). GlyRs are inhibitory neurotransmitter receptors found chiefly in the brainstem and spinal cord, and are critical for motor control and the regulation of muscle tone. Loss of inhibitory control due to immune-mediated dysfunction of the glycine pathway leads to excitatory neurotransmission and hyperexcitability of motor neurons in the spinal cord and brainstem, manifesting as rigidity, myoclonus, spasms, autonomic dysfunction, and other brainstem-related symptoms.

Approximately 20% of PERM cases are demonstrated to be paraneoplastic, mostly associated with lymphoma (5–7), thymoma (6, 8), breast cancer (6, 9), lung cancer (10), and urothelial carcinoma (11). Here, we report a complex case of PERM associated with monoclonal B-cell lymphocytosis, with a background of rheumatoid arthritis with preexisting long-term methotrexate treatment, and discuss the potential pathogeneses of the neurological and haematological diseases.

## 2 Case report

A 43-year-old, previously well woman presented with a 10-month history of insidious-onset, waxing-and-waning lower limb stiffness and spasms, gait difficulty, as well as intermittent jerking movements of the lower limbs and dysphagia. Her symptoms worsened in the 2 weeks before presentation; she was unable to mobilise out of bed by herself and sustained a fall leading to left index finger fracture.

Her past medical history was only significant for seropositive rheumatoid arthritis, diagnosed at the age of 34 years, for which she has been taking methotrexate 20 mg weekly (with adequate folic acid cover) since diagnosis with good control of her inflammatory polyarthralgia. There is a family history of autoimmune disease, with her mother and sister suffering from autoimmune thyroid diseases. She was a nonsmoker and consumed minimal alcohol. She was married with no children.

Neurological examination on presentation showed bilateral lower limb rigidity with hyperreflexia and upgoing plantar responses, in the absence of any sensory or motor impairment of the lower limbs, or any upper limb and cranial nerve abnormality. There were frequent spontaneous and stimulus-sensitive myoclonus in the lower limbs (**Supplementary Video 1**), as well as painful spasms of the lower limbs. She was commenced on clonazepam and baclofen for symptomatic management of her spasms with some improvement.

Her serum biochemistry showed an elevated CK (2615 U/L), white cell count (12.6 × 10^9^/L), and C-reactive protein (90 mg/L) but her infective screen was negative. Serological testing on presentation showed a positive antinuclear antibody (1:640, speckled) and Ro-52 antibody (titre unavailable), and negative dsDNA antibody, RNP antibody, Sm antibody, rheumatoid factor, and myositis screen (Mi-2, TIF1-γ, MDA5, NXP2, SAE1, Ku, PM-Scl 100/75, SRP, EJ, OJ, Jo-1, and PL-7/12). Biochemically, there was mild hyperthyroidism (free T_4_ 21.1 pmol/L, TSH 0.32 mIU/L), with positive thyroglobulin and thyroid peroxidase antibodies and a negative TSH-receptor antibody. There was no paraprotein. Her cerebrospinal fluid (CSF) analysis showed minimal cellularity (two mononuclear cells, zero polymorph cells) and mild hyperproteinorrhachia (0.53 g/L), and her CSF pathogen PCR panel, cytology, and oligoclonal bands were negative. Limbic antibodies (NMDAR, CASPR2, LGI-1, GABA-B, DPPX, and IgLON5), antineuronal antibodies (amphiphysin, ANNA-1, ANNA-2, CV2, Ma-1, Ma-2, PCA-1, PCA-2, SOX-1, and TR), and MOG and NMO antibodies were not detected in the serum and CSF. GlyR antibody was positive in the serum (Oxford Neuroimmunology Research Group, no titre available); this was not tested in the CSF. Glutamic acid decarboxylase 65 (GAD65) antibodies were negative. A computed tomography scan of the chest, abdomen, and pelvis was negative for malignancy. Magnetic resonance imaging of the brain and whole spinal cord was unremarkable. Gastroscopy in the evaluation of her severe dysphagia only revealed hiatal hernia and Los Angeles grade B oesophagitis.

**Figure 1:**
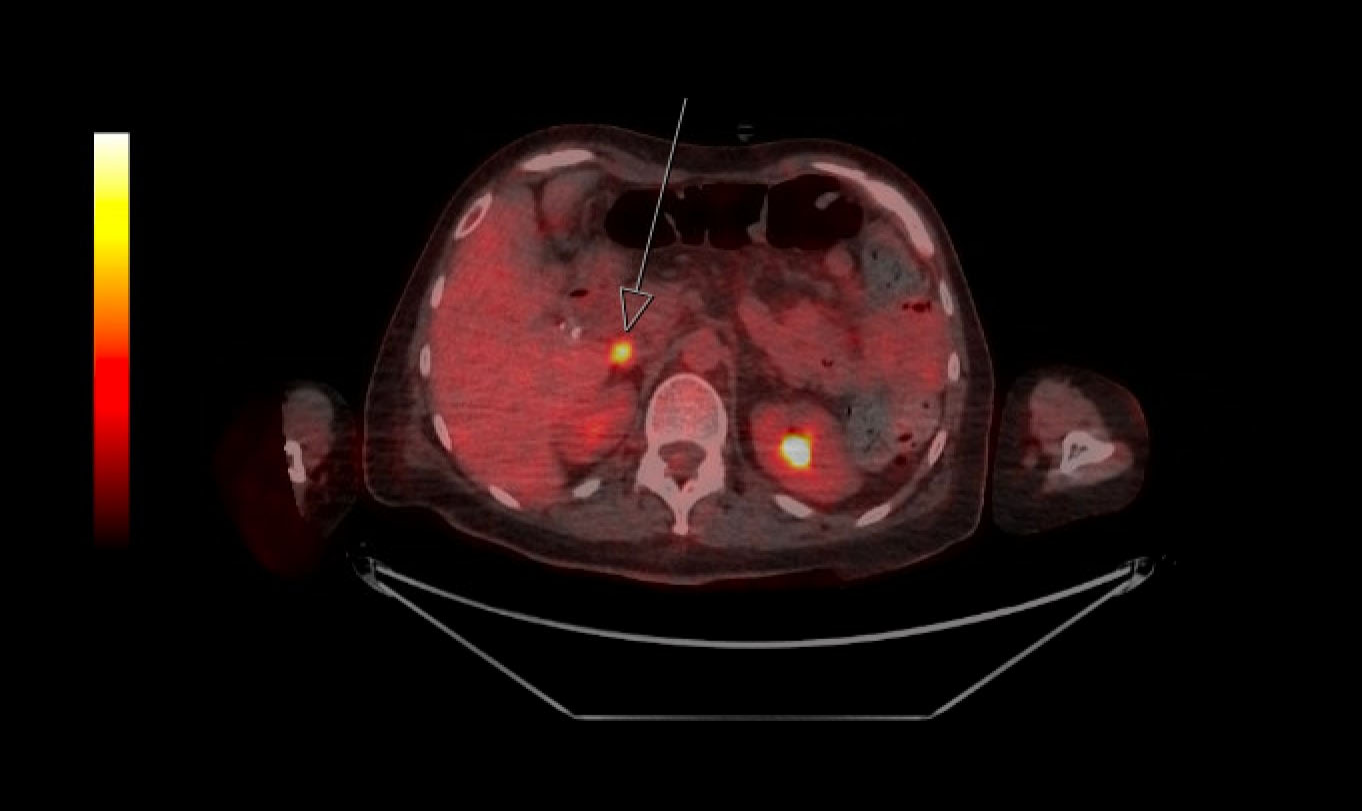
Positron emission tomography image showing isolated hypermetabolic portocaval node (depicted by arrow; SUVmax 10.0).

A diagnosis of progressive encephalomyelitis with rigidity and myoclonus (or another stiff person disorder) was made and treatment with induction intravenous immunoglobulin (IVIg) 2 g/kg was given. Her movement symptoms initially improved and she was transferred to rehabilitation, but approximately 1 month later, she relapsed with worsening myoclonus, painful spasms, and regression in rehabilitation progress. This prompted provision of further doses of maintenance IVIg and a more thorough search for an underlying malignancy. Tumour markers were normal. Her breast ultrasound showed a BI-RADS 4c lesion in the right breast, and core biopsy was negative for any neoplasm. Positron emission tomography scan revealed intense [^18^F]fluorodeoxyglucose uptake in a 17 × 11 mm portacaval lymph node (SUVmax 10.0) and no other hypermetabolic areas (**Figure 1**); endosonographic biopsy of the portacaval node unfortunately produced nondiagnostic results.

At the same time, her peripheral blood flow cytometry showed a monoclonal B-cell population (6% of lymphocytes, clone size 0.1×10^9^/L) expressing CD10, CD20, and lambda light chains, but negative for CD5 and CD19. Overall, 11.0% of cells fell within the lymphocyte gate, with T cells accounting for 83.1% of lymphocytes with a CD4:CD8 ratio of 5.9 and B cells accounting for 16.1% of lymphocytes. The immunophenotype of the abnormal clone is consistent with circulating lymphoma cells and diagnostic possibilities included small cleaved follicular lymphoma, diffuse large B-cell lymphoma of follicular centre origin, and Burkitt’s lymphoma. Bone marrow biopsy demonstrated hypocellular marrow with increased lymphocytes and small numbers of large abnormal lymphoid cells, mostly BCL2-positive; flow cytometry on the bone marrow specimen again showed a monoclonal B-cell population representing 22% of lymphocytes with the same expression profile as the serum monoclonal population. Cytogenetic studies showed the absence of *IGH::BCL2* fusion. Karyotyping was normal (46,XX)[20]. EBV-encoded RNA-1 (EBER) *in situ* hybridisation showed no EBV-positive cells in the bone marrow; Epstein–Barr virus (EBV) DNA was detected in the serum, but at a level that was too low for quantification. Thus, it appears unlikely that the monoclonal B-cell lymphocytosis was driven by EBV. The patient’s regular methotrexate was ceased due to the concern of this being a methotrexate-associated lymphoproliferative disorder.

Despite the maintenance IVIg, she went on to develop a more florid clinical syndrome with gaze palsies, diffuse brainstem myoclonus, and severe truncal and lower limb rigidity (**Supplementary Video 2**). There was also mild pyramidal-pattern weakness in the upper limbs, dysarthria, dysphonia, and involuntary expiratory vocalisations. The patient was treated with five sessions of plasmapheresis followed by repeat loading IVIg (2 g/kg) and high-dose intravenous corticosteroids (dexamethasone 8 mg daily). She responded within a few days to intensified immunosuppression; myoclonus and expiratory vocalisations were the first symptoms to improve. Her corticosteroid was changed to prednisone 25 mg daily and weaned over the next few months. She was also commenced on rituximab (375 mg/m^2^, 4 weekly doses) and mycophenolate. She underwent 1 month of inpatient rehabilitation and was able to mobilise with a single-point stick on discharge.

Her disease remained stable until a mild relapse with worsening dysphagia 3 months later, at which point the decision was made to give her extra IVIg and a top-up dose of rituximab, maintain her on 6-monthly rituximab, and cease mycophenolate. Prednisone was slowly weaned off over 10 months. IVIg was slowly weaned over 12 months. She unfortunately suffered from a low-impact trauma L1 fracture in the setting of steroid-induced osteopenia, which is now managed with zoledronic acid infusions. Otherwise, she remains well at 18 months following the initial admission, with minimal neurological symptoms and independent mobility. There is ongoing B-cell aplasia and no evidence of an active lymphoproliferative disorder whilst on maintenance rituximab. The patient’s rheumatoid arthritis remains in sustained remission following the cessation of methotrexate and initiation of rituximab as part of her long-term immunotherapy. Similarly, her thyroid function normalised on follow-up 3 months post-admission, without requiring any specific treatment for thyroid dysfunction. This suggests that her thyroid abnormality may have been transient, potentially associated with her broader autoimmune and inflammatory response.

## 3 Discussion

Here, we describe a case of severe progressive encephalomyelitis with rigidity and myoclonus with characteristic neurological findings and demonstrated seropositivity to the GlyR antibody, complicated by frequent relapses despite immunosuppression, necessitating sequential escalation in immunosuppression in order to achieve complete disease control. This corroborates growing literature data supporting the strong pathogenicity of GlyR antibodies in spinal and brainstem neurological disorders, including PERM (6), and highlights the high severity and level of disability associated with this disease and the consequent need for a timely diagnosis and institution of treatment.

A unique aspect of the case is the concurrent finding of a B-cell lymphoproliferative disorder as evidenced by a persistent circulating population of monoclonal B cells, which accounted for 6% of lymphocytes in the peripheral blood and 22% of lymphocytes in the bone marrow. This B-cell clone is CD5-negative, CD10-positive and is consistent with a non-chronic lymphocytic leukaemia-type monoclonal B-cell lymphocytosis (non-CLL-type MBL). The immunophenotype of this clone is unusual and is suggestive of post-germinal center derivation. However, there is no cytopenia, organomegaly, or lymphocytosis to suggest a full-blown lymphoma and there are no morphological features of a lymphoma on the bone marrow biopsy. There is a small hypermetabolic portocaval lymph node on PET but biopsy of this was nondiagnostic.

Whilst the significance of our patient’s non-CLL-type MBL is unknown, it has notably occurred in the setting of longstanding methotrexate use, which raises the suspicion of a methotrexate-associated lymphoproliferative disorder (MTX-LPD). MTX-LPD is a loosely defined and heterogeneous condition ranging from reactive lymphoid hyperplasia, polymorphic lymphoproliferative disorder, to lymphoid neoplasms of the diffuse large B cell or the classic Hodgkin lymphoma type (12). One possibility is iatrogenic MTX-LPD underlies the non-CLL-type MBL in our patient, which was asymptomatic at the time of her PERM diagnosis.

Literature cases now suggest that the majority of non-CLL-type MBL cases have features consistent with a marginal zone origin (13), and this is recognised in the latest (2016) update of the WHO classification for lymphoid malignancies (14). Xochelli et al. have proposed the name “clonal B-cell lymphocytosis of marginal zone origin” (CBL-MZ) to explain these diagnostically unclear cases of MBL (15). A “reactive” origin is suggested for many cases of atypical and non-CLL phenotype MBL following immune stimulation (15). If our patient’s non-CLL-type MBL is a premalignant form of marginal zone lymphoma, the isolated nodal lesion in the portocaval node is reminiscent of extranodal marginal zone lymphoma of mucosa-associated lymphoid tissue (MALT) type. Although an endoscopic examination of her stomach and duodenum during admission was normal, histopathology of the stomach was unfortunately not pursued. Interestingly, her preimmunosuppression serologies showed not only strongly positive antinuclear and anti-citrullinated peptide antibodies, but also positive Sjögren’s antibodies, making secondary Sjögren’s disease (complicating rheumatoid arthritis) possible, even though our patient did not endorse sicca symptoms. Thus, a further possibility would be that secondary Sjögren’s disease, with its chronic immune reaction and lymphomatogenic potential, underlies the non-CLL-type MBL.

Despite significant recent progress in PERM research, including the demonstration of the pathogenic role of GlyR antibodies in the disease, the aetiopathology of PERM remains largely unknown. A sizeable portion of PERM cases are paraneoplastic, especially in association with lymphoma. The coexistence of two rare pathologies (PERM and non-CLL-type MBL) in our otherwise well patient of middle age raises the possibility of causality. Our patient’s non-CLL-type MBL, which was otherwise asymptomatic, may have been a reactive phenomenon secondary to either iatrogenic methotrexate use or an underlying autoimmune disease with lymphoproliferative potential, although the autoimmune disease itself appeared inactive whilst previously under treatment with methotrexate. We postulate that the aetiology of our patient’s PERM is likely paraneoplastic secondary to this trigger, and the indolence and atypical nature of the haematological disease may have been contributed to by an efficient antitumour response that parallels the pathogenesis of her PERM, or preexisting immunosuppression (albeit mild). A similarly “subdued” manifestation of lymphoma in the context of paraneoplastic PERM/SPS is echoed by several previous cases, such as classical Hodgkin’s lymphoma with an isolated metabolically active axillary lymph node, no bone marrow infiltration or B symptoms (7), or a delayed manifestation of Hodgkin’s lymphoma shortly following treatment of PERM/SPS (5, 16, 17).

The longitudinal progression of our patient’s neurological symptoms appeared to mirror the identification of the MBL, despite the lymphoproliferative disorder remaining clinically indolent. Although the MBL did not present with overt signs such as lymphadenopathy, splenomegaly, or cytopenia, its detection at the time of worsening neurological symptoms raises the possibility of a paraneoplastic syndrome. In cases of paraneoplastic PERM, such as those associated with Hodgkin’s lymphoma, the neoplastic process may be controlled or limited, yet still trigger an autoimmune response leading to severe neurological deficits (5, 7). The temporal association between the relapse in PERM symptoms and the discovery of the hypermetabolic lymph node on PET scan further supports the hypothesis of paraneoplastic PERM in this case. Although the lymphoproliferative disorder remained asymptomatic and lacked aggressive features, it may have nonetheless contributed to the autoimmune pathology seen in PERM through a tumor-triggered immune response. Rituximab was used as the main long-term immunomodulatory therapy in our case due to its ability to target PERM (18), B-cell lymphoproliferative disorders, and rheumatoid arthritis. It has shown excellent efficacy in suppressing all the abovementioned pathologies, and has allowed the weaning of other immunosuppressive therapies, minimising therapy-associated long-term side effects.

This case is novel on multiple fronts (PERM, non-CLL-type MBL, and methotrexate use) and highlights the need for further research to elucidate the mechanism by which PERM arises in the setting of malignant and autoimmune diseases.

## 4 Conclusion

This case demonstrates a novel association between PERM, MBL, and longstanding methotrexate use, expanding our understanding of PERM’s potential paraneoplastic and autoimmune origins. The patient’s clinical course underscores the complexity of managing PERM in the context of coexisting lymphoproliferative disorders and highlights the need for ongoing research to better understand the pathogenesis and optimal treatment strategies.

## Data Availability

The original contributions presented in the study are included in the article/**Supplementary Material**. Further inquiries can be directed to the corresponding author.

## Supplementary material

Supplementary Video 1 Frequent subcortical myoclonus especially of the lower limbs.

Supplementary Video 2 Prominent brainstem signs during the patient’s inpatient relapse. Note bilateral partial ptosis, complex ophthalmoplegia with bidirectional nystagmus, dysphonia, abnormal expiratory vocalisations, and dependence on nasogastric nutrition due to dysphagia.
